# Patient Satisfaction with Nursing Care Quality: Sociodemographic, Hospitalization, and Personality Factors

**DOI:** 10.3390/nursrep16050169

**Published:** 2026-05-15

**Authors:** Marin Mamić, Ivana Mamić, Nikolina Farčić, Robert Lovrić, Ivana Barać, Željko Mudri, Marija Barišić, Željka Dujmić, Zrinka Puharić, Ivan Vukoja

**Affiliations:** 1 General County Hospital Požega, Osječka 107, 34000 Požega, Croatia; ivan.vukoja@pozeska-bolnica.hr; 2 Faculty of Dental Medicine and Health Osijek, Josip Juraj Strossmayer University of Osijek, Crkvena 21, 31000 Osijek, Croatia; nfarcic@fdmz.hr (N.F.); rlovric@fdmz.hr (R.L.); ibarac@fdmz.hr (I.B.); zmudri@fdmz.hr (Ž.M.); mbarisic@fdmz.hr (M.B.); zeljka.dujmic@bolnicasb.hr (Ž.D.); zpuharic@vub.hr (Z.P.); 3 Department od Neurosurgery, University Hospital Centre Osijek, Josip Huttler Street 4, 31000 Osijek, Croatia; 4 Department of Nursing, University of Applied Sciences in Bjelovar, 43000 Bjelovar, Croatia; 5 Faculty of Medicine, Josip Juraj Strossmayer University of Osijek, J. Huttlera 4, 31000 Osijek, Croatia; 6 Slavonski Brod General Hospital, Ulica Andrije Štampara, 35000 Slavonski Brod, Croatia; 7 Faculty of Medicine, University of Rijeka, Braće Branchetta 20, 51000 Rijeka, Croatia

**Keywords:** personality traits, hospitalization, nursing, nursing care quality, patient satisfaction

## Abstract

**Introduction/Objective**: Patient satisfaction with nursing care quality is an important patient-reported indicator of hospitalization experience. Previous studies have mainly examined sociodemographic, clinical, and organizational factors, while personality traits have rarely been included in explanatory models. This study examined the association of sociodemographic characteristics, hospitalization-related variables, and personality traits with patient satisfaction. **Methods**: A single-center cross-sectional study was conducted among hospitalized patients in a general hospital in Croatia. Data were collected at discharge using a demographic and hospitalization questionnaire, the NEO Five-Factor Inventory, and the Croatian version of the Patient Satisfaction with Nursing Care Quality Questionnaire. Group differences were analyzed using non-parametric tests, and hierarchical regression analysis was performed. **Results**: Younger age, employment, male gender, and better self-rated health were associated with higher satisfaction. Patients admitted on a scheduled basis and those staying alone or with one other person in the room were more satisfied. Sociodemographic variables explained 21.5% of the variance in satisfaction (R^2^ = 0.215; adjusted R^2^ = 0.168). After hospitalization-related variables were added, the explained variance increased to 30.1% (R^2^ = 0.301; adjusted R^2^ = 0.232). The addition of personality traits further increased the explained variance to 45.6% (R^2^ = 0.456; adjusted R^2^ = 0.385). In the final model, staying with two or more persons was negatively associated with satisfaction, whereas agreeableness and conscientiousness were positively associated with satisfaction. **Conclusions**: Patient satisfaction with nursing care quality was associated with patient characteristics, hospitalization conditions, and personality traits. Accommodation conditions and individual psychological differences should be considered when interpreting satisfaction as an indicator of nursing care quality.

## 1. Introduction

Patient satisfaction is an important indicator of nursing care quality and one of the relevant outcomes of hospitalization [1,2,3,4,5]. In contemporary healthcare systems, it is viewed as both a subjective patient experience and an indicator of care quality. Assessing patient satisfaction helps identify areas requiring organizational and professional improvement.

In nursing, satisfaction with nursing care quality is of particular importance because nurses spend the most time with hospitalized patients, and the quality of their care significantly shapes the overall treatment experience. Patient satisfaction with nursing care quality is commonly defined as the relationship between expected and received care, that is, the degree of congruence between patients’ expectations and their perception of actual care experience [6,7,8]. This suggests that patient satisfaction reflects both care characteristics and patients’ subjective interpretation of hospitalization.

Previous research has shown that patients largely base their assessment of nursing care quality on interpersonal and organizational features of care, including kindness, respect, accessibility, responsiveness, quality of information, empathy, and nurses’ professional competence [9,10,11,12]. At the same time, patient satisfaction has been associated with sociodemographic, clinical, and hospitalization-related characteristics, such as age, sex, marital status, health status, and hospital environment [10,11,12,13]. These findings confirm that patient satisfaction with nursing care quality is a complex construct resulting from the interaction of patient characteristics, hospitalization experience, and care characteristics.

A previous Croatian validation study of the PSNCQQ-Cro confirmed the reliability and validity of this instrument [14]. It also showed that satisfaction with nursing care quality may differ according to gender, age, education level, and self-rated health. Female patients, older patients, those with lower education, and those with poorer self-rated health reported lower satisfaction. These findings suggest that patient characteristics should be considered when interpreting satisfaction results. In particular, education level may be relevant as it is associated with health literacy and patients’ ability to understand, evaluate and communicate about health services. Patients with lower levels of education may have different expectations and a different level of understanding of the care process, which may affect their assessment of the quality of nursing care. Similarly, age, marital status, employment status, and self-rated health status may reflect differences in social support, functional needs, prior exposure to health services, and expectations during hospitalization. Therefore, these variables should be considered as important baseline patient characteristics when examining satisfaction with the quality of nursing care.

Factors related to hospitalization may also contribute to patient satisfaction because they reflect the immediate context in which nursing care is provided. Mode of admission, length of hospitalization, previous hospitalizations, and room occupancy may affect patients’ sense of control, privacy, and comfort. Emergency admission is often associated with uncertainty and stress, whereas planned admission may allow for better preparation and more realistic expectations [15]. Likewise, accommodation conditions, particularly the number of patients sharing a room, may influence perceptions of privacy, dignity, rest, communication with nurses, and the overall hospitalization experience [16,17,18,19,20].

Although sociodemographic, clinical, and organizational characteristics have often been investigated as predictors of patient satisfaction, the contribution of patients’ psychological characteristics has remained much less clear. This particularly applies to personality traits, which may influence emotions, interpretation of situations, and interpersonal relationships. Within the five-factor model of personality, conscientiousness, agreeableness, extraversion, neuroticism, and openness to experience represent key dimensions of individual differences that may also be relevant in nursing care contexts [21,22]. Because nursing care is highly interpersonal, it is reasonable to assume that personality traits may play an important role in the way patients experience and evaluate the care they receive [14,23,24].

Self-rated health status is also important because it can influence both the need for nursing care and the way patients interpret the hospitalization experience. Patients with poorer self-rated health may require more intensive support, have higher expectations, and be more sensitive to possible shortcomings in care organization and communication. At the same time, self-rated health is a subjective measure and may partly reflect broader individual tendencies in perception and evaluation, which further supports the inclusion of personality traits in the model.

In addition, previous research by Mamić et al. has already pointed to the relevance of personality traits in explaining healthcare-related outcomes, including job satisfaction among nurses and the association between personal characteristics and satisfaction with nursing care [24,25]. Likewise, Farčić et al. emphasized the importance of personality traits in nursing-related decision-making processes, suggesting that personality characteristics may also be relevant when examining patients’ perceptions of care [23].

According to the available literature, previous studies have mainly focused on sociodemographic, clinical, and organizational determinants of patient satisfaction, whereas psychological characteristics have been less frequently integrated into explanatory models of satisfaction with nursing care quality. In particular, there is a lack of studies that simultaneously examine sociodemographic characteristics, hospitalization-related variables, and personality traits in a single model. This gap is important because patient satisfaction may reflect not only the quality and organization of care, but also patients’ expectations, health-related needs, previous experiences, and stable individual differences in perception and interpretation.

The aim of this study was to examine the association of sociodemographic characteristics, hospitalization-related variables, and personality traits with patient satisfaction with nursing care quality. In addition, the study aimed to assess whether personality traits provide additional explanatory value beyond sociodemographic and hospitalization-related variables in a hierarchical regression model.

Based on previous research, it was expected that patient satisfaction would be associated with selected sociodemographic characteristics, including age, sex, education level, marital status, employment status, and self-rated health. It was also expected that hospitalization-related variables, particularly mode of admission, length of hospitalization, previous hospitalizations, and room occupancy, would be associated with satisfaction. Finally, it was expected that personality traits, particularly agreeableness, conscientiousness, and neuroticism, would contribute additional explained variance in patient satisfaction after controlling for sociodemographic and hospitalization-related factors.

## 2. Materials and Methods

### 2.1. Research Design

The study was conducted as a cross-sectional observational study and is presented in accordance with the STROBE guidelines for cross-sectional studies [26].

### 2.2. Participants and Study Setting

The study was conducted at the Požega General County Hospital among patients hospitalized in all hospital departments (gynecology, general surgery, abdominal surgery, traumatology, urology, ophthalmology, otolaryngology, neurology, psychiatry, infectious disease department, gastroenterology, pulmonology, cardiology, general internal medicine, maternity ward, intensive care unit). Data were collected in the period from June 2022 to February 2024.

A total of 230 patients initially participated in the study. After reviewing the collected questionnaires, 18 questionnaires were excluded from the analysis because they were incompletely or incorrectly completed. The final sample for the analysis consisted of 212 patients. The sample included 111 women (52.4%) and 101 men (47.6%). A total of 126 participants had completed secondary education (59.4%), 144 were married or cohabiting (67.9%), and 115 lived in a rural area (54.2%). The majority of respondents assessed their health status as very good, that being 79 of them (37.3%). The median age of the respondents was 54.5 years (interquartile range 36–65).

Respondents were included by convenience sampling among patients who met the inclusion criteria during the study period. Patients who met the inclusion criteria were invited to participate upon discharge from the hospital.

Patients were eligible if they were 18 years or older, understood the Croatian language, were being discharged from hospital, voluntarily agreed to participate, and had preserved motor and cognitive abilities that allowed independent completion of the questionnaire [27]. Patients younger than 18 years, those unable to understand the Croatian language, those not being discharged at the time of questionnaire administration, and those with health, motor, or cognitive limitations preventing independent questionnaire completion were excluded.

Patients who were unable to complete the questionnaire independently were excluded to preserve the self-report nature of the measurement instruments and reduce the influence of other people on the responses.

The minimum required sample size was calculated using the G*Power, version 3.1.9.7 (Heinrich Heine University Düsseldorf, Düsseldorf, Germany) [28]. For a hierarchical regression analysis with 29 predictors, a significance level of 0.05 and a power of 0.80, the minimum required sample size was 184 subjects. To detect a medium-sized effect in the differences between five independent groups, with a significance level of 0.05 and a power of 0.80, the minimum required sample size was 200 subjects. To detect a medium-sized effect in the associations between numerical variables, the minimum required sample size was 67 subjects [28,29].

### 2.3. Data Collection Procedure

Data were collected upon discharge from the hospital. Before inclusion, the purpose and procedure of the study were explained to all respondents and it was emphasized that participation was voluntary and that they could withdraw at any time without consequences for further treatment.

Data were collected by members of the research team. The questionnaires were not distributed or collected by nurses working in the departments where the patients were hospitalized, nor by persons directly involved in their daily care. This procedure was used to reduce pressure on patients and minimize socially desirable responses. It was additionally emphasized to the respondents that participation or refusal to participate would have no impact on their treatment, the attitude of the health care staff towards them, or further care.

The questionnaires were completed anonymously, independently, and in paper form. Signed informed consents were collected separately from the completed questionnaires to ensure the anonymity of the participants. No personal identifying data were collected on the questionnaires.

### 2.4. Research Instruments

#### 2.4.1. Sociodemographic and Hospitalization Questionnaire

A questionnaire was developed for the research, which included data on age, gender, marital status, level of education, place of residence, employment status, financial status, length of hospitalization, mode of admission to the hospital, number of previous hospitalizations, number of people in the patient room during treatment, and self-assessment of health status. Self-assessment of health status was measured using a five-point Likert scale, with 1 indicating “very poor” and 5 indicating “excellent”.

#### 2.4.2. NEO Five-Factor Inventory

The NEO Five-Factor Inventory consists of 60 items and assesses five basic personality dimensions: neuroticism, extraversion, openness to experience, agreeableness, and conscientiousness [30,31]. Participants assess the extent to which each statement applies to them on a five-point Likert scale. Results are presented separately for each subscale, with a higher score indicating a more pronounced presence of a particular personality trait. In previous studies, Cronbach’s alpha coefficient was 0.84 for neuroticism, 0.72 for extraversion, 0.58 for openness, 0.66 for agreeableness, and 0.80 for conscientiousness [32,33]. In the present sample, Cronbach’s alpha coefficients were 0.79 for neuroticism, 0.80 for extraversion, 0.71 for openness, 0.64 for agreeableness, and 0.77 for conscientiousness.

#### 2.4.3. Patient Satisfaction with Nursing Care Quality Questionnaire

Satisfaction with the quality of nursing care was assessed using the Croatian version of the PSNCQQ-Cro questionnaire [14]. This study used the first part of the questionnaire, which consists of 19 items aimed at assessing the quality of nursing care [34]. Responses were given on a five-point Likert scale. The original coding of responses was from 1 (“excellent”) to 5 (“poor”), and for the purposes of analysis, the responses were recoded so that a higher score indicates greater satisfaction with the quality of nursing care. The total score was obtained by summing all 19 items, with a possible range of scores from 19 to 95. The Croatian version of the questionnaire showed very high reliability, with a Cronbach’s alpha coefficient of 0.97 [14]. In this study, the Cronbach’s alpha coefficient for the PSNCQQ-Cro was 0.95.

### 2.5. Statistical Analysis

Descriptive statistical methods were used to describe the distribution of the research variables. Categorical variables were presented as absolute and relative frequencies, while numerical and ordinal variables were presented as median and interquartile range. Age was treated as a continuous variable in the analyses.

The normality of the distribution was tested using the Kolmogorov–Smirnov test. Since the assumptions of normality were not met, non-parametric methods were used in comparative analyses.

The association of variables was tested using the Spearman correlation coefficient. The Mann–Whitney U test was used to compare two independent groups, while the Kruskal–Wallis test was used to compare three or more independent groups. After a statistically significant Kruskal–Wallis test, post hoc comparisons were performed using the Dunn test with Bonferroni correction.

Hierarchical regression analysis was used to examine factors associated with nursing care satisfaction. The total satisfaction score was used as the dependent variable. The order of inclusion of predictors was determined based on theoretical and analytical considerations. In the first step, sociodemographic variables were included in the model as the basic characteristics of the respondents. In the second step, hospitalization variables were added as characteristics of the immediate treatment environment. In the third step, personality traits were included to assess their additional contribution to the explanation of satisfaction above the previously included variables.

Categorical variables in the regression models were included using indicator (dummy) coding. The reference categories were secondary school education, married or cohabiting status, employed status, emergency admission, and staying alone in the room. Gender was coded so that men were the reference category.

Although non-parametric tests were used for bivariate analyses, hierarchical linear regression was applied because the total satisfaction score was treated as a continuous outcome variable and regression assumptions were evaluated on model residuals. Before carrying out the regression analysis, the assumptions of linearity, multicollinearity, homoscedasticity, independence of residuals, normality of residuals, and the absence of influential outliers were checked. Multicollinearity was assessed by tolerance and variance inflation factor. The independence of residuals was assessed with the Durbin–Watson statistic. Homoscedasticity and normality of residuals were assessed using graphical representations of residuals, and influential values were assessed using Cook’s distance. Changes in the explained variance between steps of the hierarchical regression were assessed using R^2^, adjusted R^2^, ΔR^2^, and the F test of model change. Analyses were conducted using the complete-case method, whereby respondents with missing data for a particular analysis were not included in that analysis.

Statistical analyses were performed with IBM SPSS Statistics, version 26.0 (IBM Corp., Armonk, NY, USA) and JASP, version 0.19.3 (JASP Team, Amsterdam, The Netherlands) [35,36]. The level of statistical significance was set at *p* < 0.05.

### 2.6. Ethical Aspects of the Research

The research was conducted in accordance with the principles of the Declaration of Helsinki and with the approval of the Ethics Committee of the Požega General County Hospital (Reg. No.: 02-7/2-2/1-4-2022; approved on 20 May 2022). Written informed consent was obtained from all participants. Participation was voluntary, and refusal to participate had no impact on the treatment, care, or attitude of the healthcare staff towards the subjects.

## 3. Results

The sample included 212 patients, of whom 111 (52.4%) were women and 101 (47.6%) were men. The median age was 54.5 years (Q1–Q3 = 36–65). Most participants, 126, had completed secondary education (59.4%), 144 were married or cohabiting (67.9%), 115 lived in rural areas (54.2%), and 133 were employed (62.7%) (Table 1).

Scheduled admission was the most common mode of admission, reported by 116 (54.7%) patients. Most patients, 115, had been hospitalized once before (54.2%), while 90 (42.5%) patients stayed in a room with two or more persons. The median length of hospitalization was 6 days (Q1–Q3 = 3–9), with a range from 1 to 31 days (Table 2).

The median overall patient satisfaction with nursing care quality was 79.5 (IQR = 69–90). Among the personality traits, conscientiousness had the highest median score, Me = 33 (IQR = 28–38), while neuroticism had the lowest median score, Me = 22 (IQR = 17–28) (Table 3).

Hospitalized men were significantly more satisfied with nursing care quality than women (Mann–Whitney U test; *p* = 0.009). A significant difference was also found according to marital status (Kruskal–Wallis test; *p* = 0.013), although post hoc comparisons did not reveal significant differences between individual pairs of groups. Employment status was also significant (Kruskal–Wallis test; *p* < 0.001), with employed patients reporting significantly greater satisfaction than retired patients (Dunn–Bonferroni test; *p* < 0.05). In addition, significant differences were found according to self-rated health status at admission (Kruskal–Wallis test; *p* < 0.001), with patients rating their health as very good or excellent being significantly more satisfied than those rating it as very poor or poor. Overall, the observed effect sizes were mostly small to moderate, indicating that sociodemographic characteristics explain only part of the variability in patient satisfaction with nursing care quality (Table 4).

Regarding hospitalization-related variables, a significant difference in satisfaction was found according to the mode of admission (Kruskal–Wallis test; *p* = 0.012), with patients admitted on a scheduled basis being more satisfied than those admitted urgently (Dunn’s test; *p* < 0.05). A significant difference was also found according to the number of patients in the room (Kruskal–Wallis test; *p* < 0.001), with patients staying alone or with one other person being significantly more satisfied than those staying with two or more patients. A significant difference was also observed according to the number of previous hospital stays (Kruskal–Wallis test; *p* = 0.021), although post hoc comparisons did not show significant differences between specific pairs of groups. Overall, the observed effect sizes were small, indicating that hospitalization-related variables contributed modestly to differences in patient satisfaction with nursing care quality (Table 5).

Patient satisfaction with nursing care quality was significantly negatively correlated with age (ρ = −0.263, *p* < 0.001) and length of hospitalization (ρ = −0.247, *p* < 0.001). This indicates that older patients and patients with longer hospital stays reported lower satisfaction with nursing care quality (Table 6).

Hierarchical regression analysis showed that the first model, including sociodemographic variables, explained 21.5% of the variance in patient satisfaction with nursing care quality (R^2^ = 0.215; adjusted R^2^ = 0.168). The addition of hospitalization-related variables in the second model significantly increased the explained variance by 8.6% (ΔR^2^ = 0.086; F-change = 3.350; *p* = 0.002). In the third model, personality traits further increased the explained variance by 15.4% (ΔR^2^ = 0.154; F-change = 10.554; *p* < 0.001). The final model explained 45.6% of the variance (R^2^ = 0.456; adjusted R^2^ = 0.385). The Durbin–Watson statistic was 1.978, indicating no relevant autocorrelation of residuals (Table 7).

In the final hierarchical regression model, room occupancy with two or more persons was negatively associated with patient satisfaction with nursing care quality (B = −8.173; *p* < 0.001). Agreeableness (B = 0.480; *p* = 0.012) and conscientiousness (B = 0.522; *p* = 0.004) were positively associated with satisfaction. Admission type, categorized as other, was also negatively associated with satisfaction (B = −10.797; *p* = 0.028), although this category included a small number of patients and should therefore be interpreted cautiously. No evidence of problematic multicollinearity was observed, as all VIF values were below 3 (Table 8).

## 4. Discussion

The results of this study show that patient satisfaction with the quality of nursing care is a multidimensional construct shaped by patient characteristics, organizational conditions of hospitalization, and individual psychological characteristics. Patients reported high overall satisfaction with the quality of nursing care. This finding is consistent with previous studies conducted with the same questionnaire, which emphasize the importance of kindness, respect, responsiveness, communication, and timely assistance in assessing the quality of nursing care [14,34,37]. Previous studies also show that effective communication, warmth, empathy, and timely response to patients’ needs are among the key elements of a positive care experience [38,39].

At the same time, high levels of satisfaction should be interpreted with caution. The literature suggests that patients may sometimes evaluate nursing care more positively than the objective quality of care would justify [40,41]. Gratitude towards healthcare professionals and socially desirable responding may contribute to higher reported satisfaction [40,41,42]. Therefore, patient satisfaction should be seen as an important, but not the only indicator of the quality of nursing care. Among the variables examined, self-rated health status stood out in particular. Patients who assessed their health status as better also reported greater satisfaction with nursing care quality. This finding was observed in the univariate analyses and in the first two steps of the hierarchical model. It is consistent with previous research [5,14,43]. Patients in poorer health may have greater care needs, more frequent contact with nursing staff, and higher expectations, which may make them more sensitive to shortcomings in care [5,24]. Conversely, patients who perceive their health as better may require less intensive care and have fewer opportunities for negative experiences during hospitalization. However, self-rated health did not remain statistically significant in the final model after including personality traits, which may indicate a partial overlap of its effect with broader individual differences in the way care is experienced and interpreted.

Differences were also found with respect to the mode of admission to hospital. Patients admitted on a planned basis were more satisfied than those admitted on an emergency basis. Although this variable did not remain a stable factor in the final model, the finding is important because it suggests the possibility that the stress and unpredictability of hospitalization shape the patient experience. Planned hospitalization may provide greater predictability and preparation, whereas emergency admission is often associated with stress and uncertainty [13,15]. One of the most striking findings concerns accommodation conditions. Patients who stayed alone or with one other person in the room were more satisfied with the quality of nursing care than those who stayed with two or more people. This finding is consistent with previous research showing that patients and their families prefer single rooms [16,17]. Such accommodation may provide greater privacy, dignity, and a quieter environment [18]. Although certain organizational shortcomings of single-bed accommodation have been described, especially in terms of workload and staff turnover [19,20], our results suggest that patients perceive accommodation conditions as an important part of the overall quality of nursing care.

However, this finding should be interpreted with caution. The number of people in a patient room may not exclusively reflect accommodation conditions, but may also be related to the organization of a particular ward, diagnosis, severity of illness, functional dependence of the patient, or other patient characteristics that were not included in this model. Therefore, although the number of people in a room remained associated with satisfaction even after controlling for included variables, the possibility of residual confounding cannot be ruled out.

Hierarchical regression analysis further clarified the relative contribution of different groups of predictors. In the first step, demographic variables explained 21.5% of the variance in satisfaction with nursing care quality (R^2^ = 0.215), with sex, marital status, and self-rated health status proving significant. After adding hospitalization-related variables in the second step, the explained variance increased to 30.1% (R^2^ = 0.301), with self-rated health status remaining significant and room occupancy additionally emerging as a significant negative predictor. These findings confirm that organizational and spatial aspects of hospitalization make an independent contribution to shaping the patient experience of nursing care.

The most substantial additional contribution to the model was observed in the third step, when personality traits were included. The total explained variance increased to 45.6% (R^2^ = 0.456), with agreeableness and conscientiousness emerging as significant positive predictors of satisfaction with nursing care quality, while staying in a room with two or more persons remained a significant negative predictor.

The positive association of conscientiousness with satisfaction with the quality of nursing care can be understood in light of the characteristics of this personality trait. Conscientious individuals are generally organized, responsible, and more likely to adhere to health recommendations, which may facilitate cooperation during treatment and contribute to a more positive experience of care [44,45]. Previous studies by Mamić et al. and Farčić et al. also support the importance of personality traits in the context of healthcare [23,24]. Similarly, agreeableness may contribute to better communication with nursing staff. People with higher levels of agreeableness are more likely to be cooperative, kind, considerate, and open to advice, which may contribute to better mutual understanding and more positive evaluations of nursing care [14,46]. Although these findings do not indicate causality, they suggest that individual psychological differences may play an important role in interpreting satisfaction ratings.

Overall, the results of this study show that patient satisfaction with the quality of nursing care results from the interaction of patient characteristics, hospitalization conditions, and personal psychological characteristics. It is particularly important that the number of people in the room remains associated with satisfaction even after controlling for personality traits, which further emphasizes the importance of organizational and spatial aspects of hospital care. At the same time, the significant contribution of agreeableness and conscientiousness indicates that satisfaction assessments do not solely reflect the objective quality of care, but also the way in which patients perceive and interpret their own experience.

### 4.1. Clinical Implications

In practical application, the results indicate the importance of accommodation planning that, when organizationally possible, reduces the number of patients in the room and increases privacy during the provision of care. It is especially important to take into account patients staying in multi-bed rooms, since it is precisely the accommodation conditions that can affect the perception of privacy, dignity, rest, and the overall hospitalization experience.

The results also support the importance of individualized communication between nurses and patients. Because satisfaction is also influenced by patient characteristics, nurses should adapt communication and emotional support to individual patient needs. This is particularly important for patients who assess their health status as worse, as they may have greater needs for support, more frequent interactions with nursing staff and higher expectations of care.

The findings also indicate the need for careful interpretation of patient satisfaction surveys. The results of such surveys should not be viewed solely as a direct indicator of the objective quality of nurses’ work, but as an outcome that reflects the interaction of the quality of care, hospitalization conditions, organizational context and individual patient characteristics. Therefore, when using satisfaction indicators in quality management systems, patient characteristics and the context in which care is provided should also be taken into account.

### 4.2. Research Limitations

This study has several limitations that should be taken into account when interpreting the results. First, the cross-sectional design does not allow causal conclusions. The results obtained should therefore be interpreted as associations, rather than as evidence of a causal effect of individual factors on patient satisfaction. Second, all data were collected using a self-report method, which opens up the possibility of subjective bias, socially desirable responding, and common measurement methods. Since the questionnaires were completed at discharge, some patients may have been more inclined to express more favorable assessments due to gratitude to staff or caution towards healthcare professionals, despite the anonymous data collection procedure and the fact that the questionnaires were not collected by nurses directly involved in their care. Third, the study was conducted in a single hospital, which may limit the generalizability of the results to other healthcare institutions, departments, and organizational contexts. Patient satisfaction may depend on local organizational and accommodation characteristics. Fourth, a convenience sample was used, and the response rate was not systematically recorded. Therefore, it is not possible to fully assess possible selection bias and response rate. In addition, 18 questionnaires were excluded from the analysis due to incomplete or incorrect completion, which may represent a minor source of selection bias. Fifth, the number of predictors and dummy-coded categories was relatively large in relation to the sample size, which may increase the risk of overfitting of the model and reduce the stability of the estimates. Therefore, the results of the hierarchical regression analysis should be interpreted with caution and confirmed in future studies on larger samples. Sixth, although important sociodemographic, hospitalization, and psychological variables were included in the model, it is possible that other factors that were not included in this study influence satisfaction, such as hospital department, diagnosis upon admission, severity of illness, patient functional dependency, previous experiences with the healthcare system, expectations prior to hospitalization, and indicators of staff workload and work organization.

## 5. Conclusions

Patient satisfaction with nursing care quality was associated with sociodemographic characteristics, hospitalization-related variables, and personality traits. In the final hierarchical regression model, staying in a room with two or more persons was negatively associated with satisfaction, whereas agreeableness and conscientiousness were positively associated with satisfaction. The inclusion of personality traits increased the explained variance, suggesting that individual psychological differences may contribute to how patients evaluate nursing care.

These findings suggest that patient satisfaction should be interpreted as a multidimensional patient-reported outcome. Accommodation conditions, privacy, and individualized communication should be considered when using satisfaction indicators to evaluate and improve nursing care quality.

## Figures and Tables

**Table 1:** Sociodemographic characteristics of the study participants (*N* = 212).

			n (%)
Gender	Female		111 (52.4)
	Male		101 (47.6)
Education level	Primary school		45 (21.2)
	Secondary school		126 (59.4)
	College/undergraduate		14 (6.6)
	University degree		27 (12.7)
Marital status	Married/cohabiting		144 (67.9)
	Single		32 (15.1)
	Divorced		12 (5.7)
	Widowed		24 (11.3)
Place of residence	Rural		115 (54.2)
	Urban		97 (45.8)
Employment status	Employed		133 (62.7)
	Unemployed		21 (9.9)
	Retired		47 (22.2)
	Student		11 (5.2)
Self-rated health	Very poor		12 (5.7)
	Poor		39 (18.4)
	Good		100 (47.2)
	Very good		49 (23.1)
	Excellent		12 (5.7)
Financial status	Very poor		9 (4.2)
	Poor		27 (12.7)
	Good		84 (39.6)
	Very good		86 (40.6)
	Excellent		6 (2.8)
	Mdn (Q1–Q3)	M ± SD	Range
Age	54.5 (36–65)	51.84 ± 17.36	18–87

Note: n—number of participants; %—percentage; Mdn—median; Q1—first quartile; Q3—third quartile.

**Table 2:** Hospitalization-related characteristics of the study participants (*N* = 212).

			n (%)
Mode of admission	Emergency		82 (38.7)
	Scheduled admission		116 (54.7)
	Transfer from another institution		5 (2.4)
	Other		9 (4.2)
Number of patients in room	Alone		42 (19.8)
	With one other person		80 (37.7)
	With two or more persons		90 (42.5)
Previous hospitalizations	Once		115 (54.2)
	Twice		53 (25.0)
	Three times		23 (10.8)
	Four times		14 (6.6)
	More than four times		7 (3.3)
	Mdn (Q1–Q3)	M ± SD	Range
Length of hospitalization	6 (3–9)	6.92 ± 6.11	1–31

Note: n—number of participants; %—percentage; Mdn—median; Q1—first quartile; Q3—third quartile.

**Table 3:** Descriptive statistics of patient satisfaction with nursing care quality and personality traits.

	Mdn (Q1–Q3)	M ± SD	Range
Overall patient satisfaction with nursing care quality	79.5 (69–90)	76.31 ± 17.14	19–95
Neuroticism	22 (17–28)	23.01 ± 8.42	3–48
Extraversion	28 (23–31.75)	27.37 ± 5.93	15–42
Openness	23 (20–27)	23.33 ± 5.61	4–51
Agreeableness	29 (25–33)	28.98 ± 6.34	12–61
Conscientiousness	33 (28–38)	33.28 ± 7.54	17–71

Note: Mdn—median; Q1—first quartile; Q3—third quartile; M—mean; SD—standard deviation.

**Table 4:** Differences in Patient Satisfaction with Nursing Care Quality According to Sociodemographic Variables (*N* = 212).

			Patient Satisfaction with Nursing Care Quality			
		n	Me (IQR)	U/H (df)	r/η^2^	*p*
Gender	Male	101	83 (72.5–90.5)	4442.0	0.179	0.009 *
	Female	111	75 (65–89)			
Education level	Primary school	45	74 (61–85)	6.374 (3)	0.016	0.095 †
	Secondary school	126	80 (69–90)			
	College/undergraduate	14	80.5 (74.25–90.25)			
	University degree	27	83 (75–93.5)			
Marital status	Married/cohabiting	144	81 (71–90)	10.775 (3)	0.038	0.013 †
	Single	32	73.5 (58.5–89.25)			
	Divorced	12	87.5 (83–92)			
	Widowed	24	72 (60.75–79.5)			
Place of residence	Rural	115	79 (69.5–90)	5388.5	0.029	0.671 *
	Urban	97	80 (68–91)			
Employment status	Employed	133	83 (73–91)	19.380 (3)	0.079	<0.001 †
	Unemployed	21	81 (69–91)			
	Retired	47	71 (58–77.5)			
	Student	11	81 (73.5–88)			
Self-rated health at admission	Very poor	12	57.5 (38–76.5)	22.716 (4)	0.089	<0.001 †
	Poor	39	72 (62–83.5)			
	Good	100	80 (69.75–88)			
	Very good	49	86 (74–95)			
	Excellent	12	90.5 (81.25–93)			
Financial status	Very poor	9	78 (61–81)	6.400 (4)	0.012	0.157 †
	Poor	27	71.5 (57–83.5)			
	Good	84	80 (71–90)			
	Very good	86	81 (71–90.25)			
	Excellent	6	85 (73.25–92.5)			

Note: n = number of participants; Me = median; IQR = interquartile range; *p* = statistical significance; r = effect size for the Mann–Whitney U test; η^2^ = effect size for the Kruskal–Wallis test; values in the test statistic column represent U for the Mann–Whitney U test and H(df) for the Kruskal–Wallis test; * Mann–Whitney U test; † Kruskal–Wallis test.

**Table 5:** Differences in Patient Satisfaction with Nursing Care Quality According to Hospitalization-Related Variables (*N* = 212).

			Patient Satisfaction with Nursing Care Quality			
		n	Me (IQR)	H (df)	η^2^	*p* *
Admission type	Emergency admission	82	75.5 (63–86.75)	10.902 (3)	0.038	0.012
	Scheduled admission	116	81.5 (73–91)			
	Transfer from another institution	5	73 (67–88)			
	Other	9	79 (39–86)			
Number of patients in the room	Alone in the room	42	86 (75–90.75)	16.041 (2)	0.066	<0.001
	With one other patient	80	83.5 (71.25–92.25)			
	With two or more patients	90	74.5 (64.25–84)			
Number of hospitalizations	Once	115	81 (72–90.5)	11.579 (4)	0.037	0.021
	Twice	53	80 (70–92)			
	Three times	23	76 (65.5–88)			
	Four times	14	70 (50.25–79.5)			
	More than four times	7	67 (48–77)			

Note: n = number of participants; Me = median; IQR = interquartile range; *p* = statistical significance; η^2^ = effect size for the Kruskal–Wallis test; values in the test statistic column represent H(df); * Kruskal–Wallis test.

**Table 6:** Correlations of age and length of hospitalization with patient satisfaction with nursing care quality (*N* = 212).

		Nursing Care Quality
Age	ρ	−0.263
	*p*	<0.001
Length of hospitalization	ρ	−0.247
	*p*	<0.001

Note: ρ—Spearman’s rank correlation coefficient; *p*—statistical significance.

**Table 7:** Summary of hierarchical regression models predicting patient satisfaction with nursing care quality (*N* = 212).

Model	Predictors Included	R	R^2^	Adjusted R^2^	ΔR^2^	F-Change	*p* for F-Change	Overall F	*p*
1	Sociodemographic variables	0.464	0.215	0.168	0.215	4.526	<0.001	4.526	<0.001
2	Sociodemographic and hospitalization-related variables	0.549	0.301	0.232	0.086	3.350	0.002	4.330	<0.001
3	Sociodemographic, hospitalization-related variables, and personality traits	0.675	0.456	0.385	0.154	10.554	<0.001	6.484	<0.001

Note: R—multiple correlation coefficient; R^2^—coefficient of determination; adjusted R^2^—adjusted coefficient of determination; ΔR^2^—change in explained variance; F-change—test of change in explained variance between hierarchical steps; *p*—statistical significance.

**Table 8:** Final hierarchical regression model predicting patient satisfaction with nursing care quality (*N* = 212).

Predictor	B	SE	95% CI for B	β	t	*p*	VIF
Constant	53.362	14.321	25.109 to 81.615	—	3.726	<0.001	—
Gender	−1.793	2.149	−6.033 to 2.447	−0.052	−0.834	0.405	1.340
Education—Primary school	3.828	2.773	−1.643 to 9.299	0.092	1.380	0.169	1.501
Education—College degree	−2.353	4.007	−10.257 to 5.552	−0.034	−0.587	0.558	1.157
Education—University degree	−2.500	3.139	−8.693 to 3.693	−0.049	−0.796	0.427	1.279
Marital status—Single	−5.301	2.947	−11.116 to 0.513	−0.111	−1.799	0.074	1.300
Marital status—Divorced	5.695	4.241	−2.673 to 14.062	0.077	1.343	0.181	1.123
Marital status—Widowed	−1.825	3.509	−8.749 to 5.098	−0.034	−0.520	0.604	1.444
Employment—Unemployed	−0.460	3.446	−7.259 to 6.338	−0.008	−0.134	0.894	1.186
Employment—Retired	1.254	3.448	−5.549 to 8.056	0.030	0.364	0.717	2.395
Employment—Student	1.270	4.586	−7.777 to 10.317	0.016	0.277	0.782	1.209
Age	−0.092	0.091	−0.272 to 0.087	−0.093	−1.015	0.311	2.862
Self-rated health	1.465	1.278	−1.056 to 3.986	0.080	1.146	0.253	1.644
Length of hospital stay	−0.232	0.212	−0.650 to 0.186	−0.069	−1.094	0.275	1.361
Number of hospitalizations	−0.687	1.028	−2.715 to 1.341	−0.043	−0.668	0.505	1.387
Admission type—Scheduled admission	−3.126	2.213	−7.491 to 1.240	−0.089	−1.413	0.159	1.347
Admission type—Transfer from another institution	−4.237	7.040	−18.126 to 9.651	−0.038	−0.602	0.548	1.334
Admission type—Other	−10.797	4.861	−20.386 to −1.207	−0.127	−2.221	0.028	1.123
Room occupancy—With one other person	0.683	2.944	−5.125 to 6.492	0.016	0.232	0.817	1.579
Room occupancy—With two or more persons	−8.173	2.210	−12.534 to −3.813	−0.236	−3.698	<0.001	1.390
Neuroticism	−0.258	0.166	−0.585 to 0.069	−0.126	−1.557	0.121	2.254
Extraversion	0.165	0.206	−0.242 to 0.571	0.057	0.799	0.425	1.729
Openness	0.182	0.182	−0.177 to 0.541	0.060	1.001	0.318	1.211
Agreeableness	0.480	0.189	0.107 to 0.853	0.177	2.540	0.012	1.663
Conscientiousness	0.522	0.181	0.164 to 0.879	0.215	2.879	0.004	1.913

Note: B = unstandardized regression coefficient; SE = standard error; CI = confidence interval; β = standardized beta coefficient; t = test statistic; *p* = statistical significance; VIF = variance inflation factor. Reference categories were secondary school education, married status, employed status, emergency admission, and staying alone in the room.

## Data Availability

The data presented in this study are available on request from the first or corresponding authors. The data are not publicly available due to privacy and ethical restrictions.

## Public Involvement Statement

No public involvement in any aspect of this research.

## Guidelines and Standards Statement

This manuscript was prepared in accordance with the STROBE guidelines for cross-sectional studies.
